# Weighted BATS Codes with LDPC Precoding

**DOI:** 10.3390/e25040686

**Published:** 2023-04-19

**Authors:** Wenyue Zhang, Min Zhu

**Affiliations:** 1 The State Key Laboratory of Integrated Services Networks, Xidian University, Xi’an 710071, China; 2 Science and Technology on Communication Networks Laboratory, Shijiazhuang 050081, China

**Keywords:** network coding, BATS codes, LDPC codes, unequal error protection, density evolution

## Abstract

Batched Sparse (BATS) codes are a type of network coding scheme that use a combination of random linear network coding (RLNC) and fountain coding to enhance the reliability and efficiency of data transmission. In order to achieve unequal error protection for different data, researchers have proposed unequal error protection BATS (UEP-BATS) codes. However, current UEP-BATS codes suffer from high error floors in their decoding performance, which restricts their practical applications. To address this issue, we propose a novel UEP-BATS code scheme that employs a precoding stage prior to the weighted BATS code. The proposed precoding stage utilizes a partially regular low-density parity-check (PR-LDPC) code, which helps to mitigate the high error floors in the weighted BATS code We derive the asymptotic performance of the proposed scheme based on density evolution (DE). Additionally, we propose a searching algorithm to optimize precoding degree distribution within the complexity range of the precoding stage. Simulation results show that compared to the conventional weighted BATS codes, our proposed scheme offers superior UEP performance and lower error floor, which verifies the effectiveness of our scheme.

## 1. Introduction

Data packets may be lost during transmission in wireless network communication due to path fading and interference. Network coding [1], which allows relay nodes to encode packets, is an effective forward error correction (FEC) code in wireless erasure networks. Random linear network coding (RLNC) [2] is a typical network coding scheme, whose coding coefficients are randomly selected over a finite field. RLNC can improve throughput effectively. However, conventional RLNC has high decoding complexity and needs big cache capacity, which restrict its application. As a new network coding scheme, the BATS code [3] has been extensively studied in recent years due to its low complexity and rateless characteristic.

Input packets are encoded in batches by the BATS code, which is composed of the outer code and the inner code. The outer code adopts the matrix form of the Luby Transform (LT) code, which is a kind of fountain codes. The BATS code is rateless because the outer code can produce any number of batches. The inner code adopts RLNC in the same batch. Designing the degree distribution function for BATS codes can effectively enhance their decoding performance when employing the belief propagation (BP) algorithm. In [3,4], the design methods of degree distribution of the BATS code are given in the case of infinite code length and finite code length, respectively. The benefit of the BATS code is that only a fixed number of packets need to be buffered and processed by the relay node for the fixed batch size of the BATS code.

In order to improve the performance of BATS codes, Zhou et al. [5] investigated the design of the inner code to maximize the expected batch transfer matrix rank normalized by the total number of packets transmitted by both the source and intermediate nodes. Juan Yang et al. [6] proposed an improved algorithm which iteratively performs BP decoding and incremental Gaussian elimination for decoding finite-length BATS codes for improving the decoding performance. To achieve lower latency, a sliding window framework was analyzed in [7], which divides the information blocks into smaller sub-blocks and jointly optimizes the degree distribution and window selection probability of each sub-window. Wang et al. [8] proposed cascading LDPC codes before BATS codes.

BATS code has great application prospects in many scenarios. Gao et al. [9] proposes a joint infrastructure-to-vehicle and vehicle-to-vehicle communication scheme using batched sparse coding to efficiently distribute content to vehicles passing by roadside units, reducing transmission delay and traffic overhead. Yeung et al. [10] discussed the potential and prospects of using BATS codes for space communication. Wang et al. [11] investigated the use of batched sparse (BATS) codes in a butterfly network for multicast communication.

Conventional BATS codes can only provide equal error protection (EEP) for all data. However, in the scenarios of image and video data transmission, some data require higher reliability than others. So unequal error protection (UEP) technology is needed. Xu et al. proposed the weighted BATS code [12] and the expanding window BATS (EW-BATS) code [13] with UEP characteristics. Xiang et al. [14] proposed the feedback expanding window BATS (FEW-BATS) code, which decreases average overhead of successful decoding in the less important packets (LIP) and has no effect on the decoding performance of the more important packets (MIP) by adding a single feedback on the basis of the EW-BATS code.

The conventional UEP-BATS codes have a high error floor. This is because BATS codes randomly select input packets during encoding, which leads a small number of input packets participating in encoding only a few times or even never participating in encoding. To solve this problem, we propose a new UEP-BATS code scheme which performs LDPC precoding before the weighted BATS code. In order to obtain better UEP, we adopt partially regular LDPC (PR-LDPC) [15] as the precoding scheme. We derive the density evolution (DE) analysis when the code proposed in this paper is decoded by BP algorithm on binary erasure channel (BEC) and optimize the relevant parameters according to the DE analysis. Finally, we verify the performance of the proposed code through simulations.

## 2. BATS Codes and Weighted BATS Codes

### 2.1. BATS Code

The BATS code consists of the outer code and the inner code. We assume that there are *K* input packets, and the set of input packets is denoted by $S=\{s_{1},s_{2},\ldots ,s_{K}\}$ where $s_{k}$ is the *k*th input packet and each packet consists of *L* bits. The outer code encodes the input packets into batches. We assume that the batch size is *M* and the degree distribution is denoted by $\Omega \left(x\right)=\sum_{d=1}^{D}\Omega_{d}x^{d}$, where $\Omega_{d}$ is the probability of generating a degree value of *d* and *D* is the maximum degree. The encoding process in *i*th batch of the BATS code is given as follows:

(1) A degree value $d_{i}$ is generated according to Ω(x).
(2) $d_{i}$ input packets in S are randomly selected to generate $S_{i}$.
(3) Batch $C_{i}=S_{i}G_{i}$ is generated, where $G_{i}$ is a random matrix with its elements on a finite field $F_{q}$ with size q and dimension $d_{i}\times M$.
(4) The inner code of the BATS code is RLNC in the batch.

Then the encoded packets are sent to the downstream node. Assuming that the transmission matrix of the *i*th batch is $A_{i}$, the packets of the *i*th batch received by the destination node are

(1) $$Y_{i}=C_{i}A_{i}=S_{i}G_{i}A_{i},$$

where $A_{i}$ has dimension $M\times {M_{i}}^{\prime }$, and ${M_{i}}^{\prime }$ denotes the number of packets received in the *i*th batch.

The number of batches generated is $n=\frac{(1+\gamma )K}{M}$ where γ is the encoding redundancy. We let $\mu =\Omega^{\prime }\left(1\right)$ and $\theta =\frac{1+\gamma }{M}\mu$ denote the average degree of output batches and input packets, respectively. $\Lambda \left(x\right)=\sum_{i}\Lambda_{i}x^{i}$ is the degree distribution of the input packets, where

(2) Λi=ni(μK)i(1−μK)n−i≈e−θθii!.

BATS codes can be decoded using the BP algorithm. In [16], the AND-OR tree analysis of BATS codes using the BP algorithm is introduced in detail. In the AND-OR tree, the OR-nodes denote the input data packets, and the AND-nodes denote the output batches. The root node of AND-OR tree is an OR-node. The children of OR-nodes are AND-nodes and the children of AND-nodes are OR-nodes. Suppose $h=[h_{1},h_{2},\ldots ,h_{M}]$ denotes the channel rank distribution and $h_{r}$ denotes the probability that the rank of the batch received by the destination node is *r*. There are the following conclusions about AND-OR tree:

- The probability that an OR-node has *i* children is $\delta_{i}=\frac{\left(i+1\right)\Lambda_{i+1}}{\theta },i=0,1,\ldots ,n-1$.
- The probability that an AND-node has *i* children is $\omega_{i}=\frac{\left(i+1\right)\Omega_{i+1}}{\mu },i=0,1,\ldots ,D-1$.
- An OR-node is decodable only when any of its children are decodable.
- An AND-node is associated with rank *r* with probability $h_{r}$ where r=1,2,…,M and it is decodable if less than r−1 children are undecodable.

The probability that the root node is undecodable in the *l*th iteration is

(3) $$\left\{\begin{array}{c} y_{0}=1,\\ y_{l}=\delta \left(1-\omega \left(1-y_{l-1}\right)\right), \end{array}\right.$$

where

(4) $$\delta \left(x\right)=\sum_{i=0}^{n-1}\delta_{i}x^{i}=e^{\theta (x-1)},$$

and

(5) ω(1−x)=μ∑d=1Dωd−1∑r=1Mhr∑j=1r−1d−1j(1−x)d−1−jxj.

### 2.2. Weighted BATS Codes

**Figure 1:**
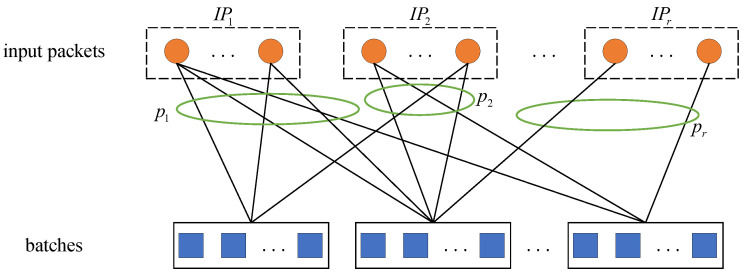
The encoding process of weighted BATS codes.

Weighted BATS codes change the probability of being selected for encoding by setting different weight factors for input packets of different importance. We assume that there are *K* input packets and they are divided into *m* important levels. The *j*th level of input packets is denoted by $IP_{j}$. The number of packets with the *j*th important level is $\alpha_{j}K$ and $\sum_{j=1}^{m}\alpha_{j}=1$. The probability of *j*th level packets being selected is $p_{j}=T_{j}/K$ where $T_{j}$ is the weight factor of the *j*th important packets. The value of $T_{j}$ has to satisfy $\sum_{j=1}^{m}\alpha_{j}T_{j}=1$ and $p_{i}>p_{j}\left(i<j\right)$. Figure 1 illustrates the encoding process of weighted BATS codes at the source node.

According to the analysis of weighted BATS codes in [12], the probability of a *j*th important packet undecodable after *l* iterations of BP is

(6) $$\begin{array}{c} \left\{\begin{array}{cc} y_{0,j} & =1,\\ y_{l,j} & =\delta^{\left(j\right)}\left(1-\omega \left(1-\sum_{u=1}^{m}\alpha_{u}T_{u}y_{l-1,u}\right)\right)\\ & =\exp[-\theta_{j}\omega \left(1-\sum_{u=1}^{m}\alpha_{u}T_{u}y_{l-1,u}\right)], \end{array}\right. \end{array}$$

where $\theta_{j}=\frac{\left(1+\gamma \right)\mu T_{j}}{M}$ is the average degree of the input packets with *j*th-level importance.

## 3. Weighted BATS Code with LDPC Precoding

### 3.1. Encoding Scheme

In the conventional weighted BATS coding scheme, the input packets are randomly selected with different probabilities when encoding. Therefore, some input packets are rarely selected or even never selected, which would lead to high error floor at the receiving side. In order to lower the packet error rate, we propose a novel UEP-BATS code, which performs PR-LDPC codes precoding before the weighted BATS code, and precoding phase can be utilized to mitigate errors and recover input packets that cannot be restored by weighted BATS codes. In our scheme, the encoding process of the source node is divided into a precoding phase and a weighted BATS code encoding phase. The encoding process of these two phases is as follows:

**Precoding phase**: The PR-LDPC code [15] with UEP characteristics is used for precoding in our coding scheme. We assume that the number of the input packets is *K*, and *N* intermediate packets are generated by precoding. Therefore, the number of parity packets (PP) is N−K, and the code rate of precoding is R=K/N. Let the precoding check matrix be $H=\left[\begin{array}{cccc} H_{1} & H_{2} & \cdots & H_{m}|H_{P} \end{array}\right]$, where $H_{j}$ (1≤j≤m) is the sub-matrix corresponding to the $IP_{j}$, $H_{P}$ is the sub-matrix corresponding to PP and $H_{P}$ is a non-singular matrix. Let $d_{j}$ be the degree of the $IP_{j}$, $d_{P}$ be the degree of PP, and $d_{C}$ be the degree of check nodes. So the column weight of $H_{j}$ is $d_{j}$, the column weight of $H_{P}$ is $d_{P}$, and the row weight of H is $d_{C}$. According to the analysis in [15], the decoding error probability of the $IP_{i}$ is lower than that of the $IP_{j}$ when $d_{i}>d_{j}$. Since PP are not included in the packets to be recovered, $d_{1}>d_{2}>\cdots >d_{m}>d_{P}$ should be satisfied when $d_{C}$ is determined. The input packets are denoted by $B=\left[\begin{array}{cccc} B_{1} & B_{2} & \cdots & B_{m} \end{array}\right]$, where $B_{j}$ denotes the sub-matrix formed by the $IP_{j}$, so the dimension of $B_{j}$ is $L\times \alpha_{j}K$. The intermediate packets are denoted by $B^{\prime }=\left[\begin{array}{c} B|B_{P} \end{array}\right]$, where $B_{P}$ is the sub-matrix formed by PP, so the dimension of $B_{P}$ is L×(N−K). According to $H\cdot {\left(B^{\prime }\right)}^{T}=0$, we can obtain

(7) $$H_{1}\cdot B_{1}^{T}+H_{2}\cdot B_{2}^{T}+\cdots +H_{m}\cdot B_{m}^{T}+H_{P}\cdot B_{P}^{T}=0.$$

The precoding process is performed on the finite field GF(2), so the PP generated by precoding is

(8) $$B_{P}=\left(B_{1}\cdot H_{1}^{T}+B_{2}\cdot H_{2}^{T}+\cdots +B_{m}\cdot H_{m}^{T}\right)\cdot {\left(H_{P}^{T}\right)}^{-1}$$

**Weighted BATS phase**: Weighted BATS coding is performed on the intermediate packets in this phase. The intermediate packets are divided into *m* levels, where the *i*th level of intermediate packets are consistent with the *i*th level of input packets, 1≤i≤m−1, and the *m*th level of intermediate packets includes the *m*th level of input packets and the PP. Therefore, the proportion of intermediate packets of *i*th level is $\alpha_{i}^{\prime }=\alpha_{i}K/N=\alpha_{i}R$, and the proportion of intermediate packets of *m*th level is $\alpha_{m}^{\prime }=\left(\alpha_{m}K+N-K\right)/N=\alpha_{m}R+\left(1-R\right)$. The weight factor set for the *j*th level of intermediate packets is $T_{j}$ where 1≤j≤m. Therefore, when weighted BATS coding is performed on the intermediate packet, the probability that the *j*th level of the intermediate packet is selected is $p_{j}=T_{j}/N$.

**Figure 2:**
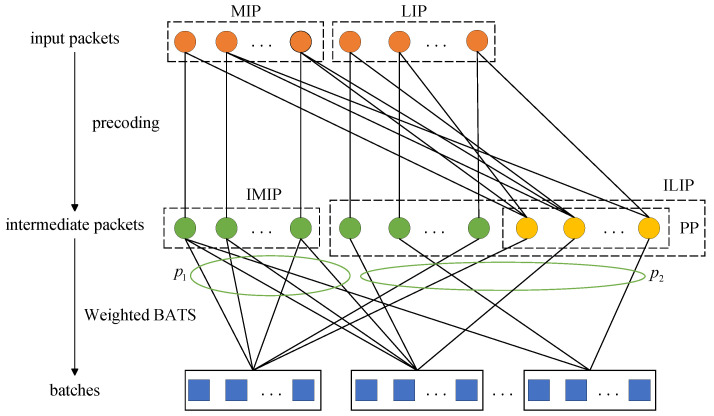
The encoding process of weighted BATS code with precoding.

Figure 2 illustrates the encoding procedure of weighted BATS codes based on PR-LDPC precoding at the source node with m=2 as an example. Input packets can be classified as MIP and LIP. Intermediate packets can be classified as MIP, LIP, and PP, or can be further classified as IMIP (Intermediate more important packets) and ILIP (Intermediate less important packets).

### 3.2. Decoding Scheme

**Figure 3:**
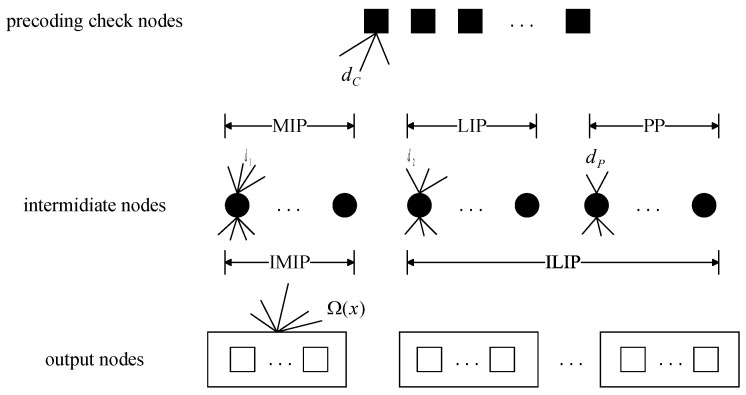
Bipartite graph of the weighted BATS code with PR-LDPC precoding.

At the destination node, The receiver uses the BP algorithm for decoding and the bipartite graph of the BP algorithm is shown in Figure 3. The decoder of receiver consists of a weighted BATS code decoder and a precoding decoder. The specific decoding processes of these two decoders are as follows:

**Weighted BATS decoder**: Similar with the BP algorithm in the conventional BATS codes, each output node in Figure 3 corresponds to a batch of weighted BATS codes. Due to the packet loss in the transmission, the number of receiving packets in the *i*th batch $M_{i}^{\prime }\leq M$. The rank of the *i*th batch is $r_{i}=rank\left(G_{i}A_{i}\right)$. Let $d_{i}$ and $S_{i}$ denote the degree and the input packets set of *i*th batch, respectively. If $r_{i}=d_{i}$, $S_{i}$ can be decoded by Gaussian elimination and delete all edges connected to the intermediate nodes corresponding to $S_{i}$ in the bipartite graph. If the kth(k≠i) batch contains intermediate node $s_{i}\left(s_{i}\in S_{i}\right)$, remove the row corresponding to $s_{i}$ in $G_{k}$. Repeat the process until no batch satisfies that the degree is equal to the rank.

**Precoding decoder**: In each iteration, the precoding check nodes with degree of 1 are decodable. If the degree of *j*th check node is 1 and it is connected only to intermediate node $s_{j}$, the input packet corresponding to $s_{j}$ is decodable. Then all edges connected to $s_{j}$ in the graph are deleted. If $s_{j}$ is selected to encode the *k*th batch, the row corresponding to $s_{j}$ in $G_{k}$ is removed. The process is repeated until there is no check node with degree of 1.

**Figure 4:**
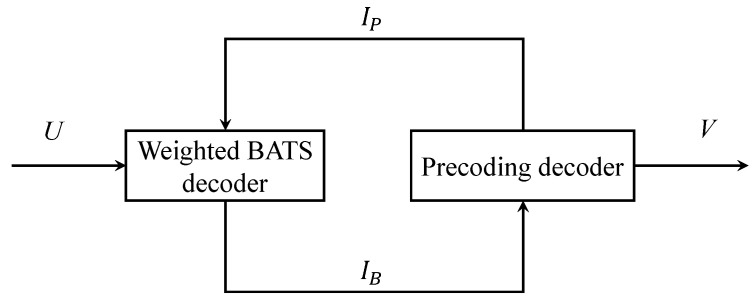
The decoding process of the of the weighted BATS code with LDPC precoding.

The decoding process is shown in Figure 4, where *U* and *V* represent the index of the received packets and recovered packets, respectively. The receiver side performs the weighted BATS decoder and the precoding decoder in a cyclic manner. At the beginning of each cycle, the sets $I_{B}$ and $I_{P}$ are initialized as empty sets. Subsequently, the weighted BATS code decoding and the precoding decoding are executed. The index of the intermediate data packets decoded by weighted BATS decoder and precoding decoder are added to the sets $I_{B}$ and $I_{P}$, respectively. The intermediate packet indexes in sets $I_{B}$ and $I_{P}$, but not belonging to PP, are inserted into set *V*. The receiver side restarts weighted BATS decoding because there may still exists some decodable batches after parts of the intermediate data packets are decoded by precoding decoder. The decoding process is stopped when set $I_{B}$ is empty.

## 4. Performance Analysis and Parameter Optimization

### 4.1. Decoding Error Probability

In this subsection, we use the AND-OR tree analysis to derive the DE formulas of the weighted BATS code with LDPC precoding under BP decoding on BEC according to the conclusions in [15,17]. During the BP decoding process, erasure information is repeatedly passed between precoding check nodes, intermediate nodes, and output nodes. Each node updates its information upon receiving information from other nodes and passes the updated information to other nodes. Let ${\bar{\Lambda }}^{\left(j\right)}\left(x\right)=\sum_{i}{\bar{\Lambda }}_{i}^{\left(j\right)}x^{i}$ denotes the input degree distributions of *j*th intermediate nodes, and ${\bar{\delta }}^{\left(j\right)}\left(x\right)=\sum_{i}{\bar{\delta }}_{i}^{\left(j\right)}x^{i}$ represents the degree distribution of edges connected to *j*th intermediate nodes. So we can obtain ${\bar{\delta }}^{\left(j\right)}\left(x\right)={\bar{\Lambda }}^{{\left(j\right)}^{\prime }}\left(x\right)/{\bar{\Lambda }}^{{\left(j\right)}^{\prime }}\left(1\right)$. Let *E* be the set of edges between the intermediate nodes and the precoding check nodes. Let $\lambda_{j}$ and $\lambda_{P}$ respectively denote the probability of selecting an edge from *E* at random, which corresponds to the *j*th level input packets and PP. So we have

(9a) $$\begin{array}{cc} \lambda_{j} & =\frac{\alpha_{j}Kd_{j}}{\left|E\right|}, \end{array}$$

(9b) $$\begin{array}{cc} \lambda_{P} & =\frac{\left(1-R\right)Nd_{P}}{\left|E\right|}, \end{array}$$

where $\left|E\right|=\sum_{j=1}^{m}\alpha_{j}Kd_{j}+\left(1-R\right)Nd_{P}$, indicating the number of edges between the intermediate nodes and the precoding check nodes.

At *i*th BP iteration, let $P_{j,i}$ and $P_{P,i}$ respectively denote the probability of passing erasure information to the precoding check nodes from the intermediate nodes corresponding to $IP_{j}$ and PP; $Q_{j,i}$ and $Q_{P,i}$ respectively denote the probability of passing erasure information to the output nodes from the corresponding intermediate nodes to $IP_{j}$ and PP; $U_{i}$ denotes the probability of passing erasure information to the intermediate nodes from the precoding check nodes; $V_{i}$ denotes the probability of transmitting erasure information to the intermediate nodes from the output nodes. Subsequently, the respective mathematical expressions for the aforementioned probabilities will be presented.

When i=0, all intermediate nodes are initialized to 1, which means that $P_{j,0}=P_{P,0}=1$. When i≥1, $P_{j,i}$ is equivalent to the probability that the intermediate nodes corresponding to $IP_{j}$ receive erasure messages from the other $d_{j}-1$ edges connected to the precoding check nodes and all edges connected to the output nodes. The former probability is ${\left(U_{i-1}\right)}^{d_{j}-1}$, and the latter probability is $\sum_{i}{\bar{\Lambda }}_{i}^{\left(j\right)}{\left(V_{i-1}\right)}^{i}={\bar{\Lambda }}^{\left(j\right)}\left(V_{i-1}\right)$. Therefore, the formula for calculating $P_{j,i}$ is

(10) $$P_{j,i}={\left(U_{i-1}\right)}^{d_{j}-1}{\bar{\Lambda }}^{\left(j\right)}\left(V_{i-1}\right).$$

Similarly, the formula for calculating $P_{P,i}$ is

(11) $$P_{P,i}={\left(U_{i-1}\right)}^{d_{P}-1}{\bar{\Lambda }}^{\left(m\right)}\left(V_{i-1}\right).$$

When i=0, all intermediate nodes are initialized to 1, which means that $Q_{j,0}=Q_{P,0}=1$. When i≥1, $Q_{j,i}$ is equivalent to the probability that the intermediate nodes corresponding to $IP_{j}$ receives the erasure messages from other output nodes and all precoding check nodes. The former probability is ${\left(U_{i-1}\right)}^{d_{j}}$, and the latter probability is equivalent to the probability that all children of the intermediate node in the AND-OR tree pass the erasure messages to it. So we have

(12) $$Q_{j,i}={\left(U_{i-1}\right)}^{d_{j}}{\bar{\delta }}^{\left(j\right)}\left(V_{i-1}\right).$$

Similarly, we can calculate $Q_{P,i}$ by

(13) $$Q_{P,i}={\left(U_{i-1}\right)}^{d_{P}}{\bar{\delta }}^{\left(m\right)}\left(V_{i-1}\right).$$

The probability that the precoding check node passes on the erasure message to the intermediate node is denoted as $U_{i}$, which is equivalent to the probability that the check node receives the erasure messages from other $d_{C}-1$ intermediate nodes, so

(14) $$U_{i}=1-{\left(1-\sum_{j=1}^{m}\lambda_{j}P_{j,i}-\lambda_{P}P_{P,i}\right)}^{d_{C}-1}.$$

The probability that the output node passes on the erasure message to the intermediate node is denoted as $V_{i}$

(15) $$V_{i}=1-\omega \left(1-y\right),$$

where *y* denotes the probability that a child node of the AND node in the AND-OR tree transmits an erasure message, ω(1−y) denotes the probability that less than r−1 child nodes transmit erasure messages to the AND node, and *r* is the rank of the coding coefficient matrix corresponding to the AND node, so

(16) $$y=\sum_{j=1}^{m}\alpha_{j}RT_{j}Q_{j,i}+\left(1-R\right)T_{m}Q_{P,i},$$

and

(17) ω(1−y)=μ∑d=1Dωd−1∑r=1Mhr∑j=1r−1d−1j(1−y)d−1−jyj.

The above is the calculation process for the probability of erasure message transmitted between nodes. Based on these probabilities, we can make an asymptotic estimate of the decoding error probabilities of $IP_{j}$ which is given by:

(18) $$P_{i}^{\left(j\right)}=\frac{d_{j}P_{j,i}+{\bar{\theta }}_{j}Q_{j,i}}{d_{j}+{\bar{\theta }}_{j}},$$

where ${\bar{\theta }}_{j}=n\mu p_{j}$ is the average degree of the intermediate packets with *j*th level importance.

### 4.2. Complexity

In the conventional weighted BATS code, the M encoded packets in the batch is generated by linearly combining μ input packets on average. Therefore, the encoding complexity of generating *n* batches in the conventional weighted BATS code is O(nμLM). The encoding complexity is slightly elevated in our scheme due to the addition of the precoding phase. During the precoding phase, the encoding complexity is related to the row weight $d_{C}$ of the parity matrix and the number of parity packets. A packet XOR operation must be performed during encoding for each element in the parity matrix with a value of “1”. Therefore, the encoding complexity in the precoding phase is $O(Ld_{C}\left(N-K\right))$. The additional complexity introduced in the precoding phase is constant and independent of the number of batches generated.

At the destination node, the decoding process is divided into two stages, where the average complexity of the weighted BATS decoder is $O(nM^{3}+n\mu LM)$, and the complexity of the precoding decoder is $O(Ld_{C}\left(N-K\right))$. Therefore, the decoding complexity of this scheme is $O(nM^{3}+n\mu LM+Ld_{C}\left(N-K\right))$.

In summary, compared to conventional weighted BATS codes, our scheme only add a marginal and fixed increase in the complexity of the encoding process and decoding process.

### 4.3. Optimization of Precoding Degree Distribution

This subsection proposes an optimization algorithm for the precoding degree distribution under the constraint of limited precoding complexity. Based on the complexity analysis presented in the previous subsection, it can be concluded that, under the assumption of data packet length *L* and precoding rate *R*, the complexity of the precoding phase is solely determined by the row weight $d_{C}$ of the parity check matrix H. Therefore, the limitation of complexity in the precoding phase refers to its complexity being constrained by the maximum value of $d_{C}$. In other words, the maximum allowable value of $d_{C}$ restricts the complexity of this phase. So we need to find the optimal values of $d_{j}$ and $d_{P}$ in the case where $d_{C}$ is fixed.

According to the fact that the sum of the number of “1” elements in all columns of the parity check matrix H is equal to the sum of the number of “1” elements in all rows, we can obtain

(19) $$\sum_{j=1}^{m}\alpha_{j}Rd_{j}+\left(1-R\right)d_{P}=\left(1-R\right)d_{C}.$$

Suppose that $d_{j}$ and $d_{P}$ have lower bounds of 1, and $D_{j}$ and $D_{P}$ denote the maximal values of $d_{j}$ and $d_{P}$, respectively. Due to $d_{1}>d_{2}>\cdots >d_{m}>d_{P}$, we sequentially determine $d_{1}$, $d_{2}$, ⋯, $d_{m}$ and $d_{P}$. According to (19), $D_{j}$ and $D_{P}$ can be calculated using the following methodology.

(1) When j=1, it can be inferred that the variable $d_{1}$ reaches its maximum value under the circumstance of $d_{2}=d_{3}=\cdots =d_{m}=d_{P}=1$, so

(20) $$D_{1}=\frac{\left(1-R\right)d_{C}-\sum_{i=2}^{r}\alpha_{i}R-\left(1-R\right)}{\alpha_{j}R}.$$

(2) When 2≤j≤m, it can be inferred that the variable $d_{j}$ reaches its maximum value under the circumstance of $d_{j+1}=d_{j+2}=\cdots =d_{m}=d_{P}=1$, so

(21) $$D_{j}=min\left\{d_{j-1}-1,\frac{\left(1-R\right)d_{C}-\sum_{i=1}^{j-1}\alpha_{i}Rd_{i}-\sum_{i=j+1}^{m}\alpha_{i}R-\left(1-R\right)}{\alpha_{j}R}\right\}.$$

(3) When $d_{j}$ is completely determined, the maximum possible value of $d_{P}$ is

(22) $$D_{P}=min\left\{d_{m}-1,\frac{\left(1-R\right)d_{C}-\sum_{i=1}^{m}\alpha_{i}Rd_{i}-\left(1-R\right)}{1-R}\right\}.$$

Given the aforementioned expression, upon specifying the value of $d_{C}$, one can determine the values of $D_{1}$, $D_{2}$, ⋯, $D_{m}$ and $D_{P}$ in a sequential manner. Thereafter, the search process can be employed to obtain the optimal precoding degree distribution, as presented in Algorithm 1, where the symbol “←” signifies assignment.
**Algorithm 1** Precoding degree distribution optimization algorithm**Input:** *K*, $\alpha_{1},\cdots ,\alpha_{m}$, *R*, Ω(x), $d_{C}$ **Output:** $d_{1}$, ⋯, $d_{m}$, $d_{P}$ 1:$P_{1}\leftarrow 1$, $P_{2}\leftarrow 1$, ⋯, $P_{m}\leftarrow 1$; 2:Calculate $D_{1}$ based on (20); 3:**for** $d_{\left(1\right)}=1$ to $D_{1}$ **do** 4: Calculate $D_{2}$ based on (21); 5: **for** $d_{\left(2\right)}=1$ to $D_{2}$ **do** 6:  ⋮ 7:  Calculate $D_{m}$ based on (21); 8:  **for** $d_{\left(m\right)}=1$ to $D_{m}$ **do** 9:   Calculate $D_{P}$ based on (22);10:   **for** $d_{p}=1$ to $D_{P}$ **do**11:    Calculate the decoding error probability $P^{\left(j\right)}$ for the $IP_{j}$ based on (18);12:    **if** $P^{\left(1\right)}<P_{1}$ && ⋯ && $P^{\left(m\right)}<P_{m}$ && $P^{\left(P\right)}<P_{P}$ **then**13:     $P_{1}\leftarrow P^{\left(1\right)}$, ⋯, $P_{m}\leftarrow P^{\left(m\right)}$;14:     $d_{1}\leftarrow d_{\left(1\right)}$, ⋯, $d_{m}\leftarrow d_{\left(m\right)}$, $d_{P}\leftarrow d_{p}$;15:    **end if**16:   **end for**17:  **end for**18:  ⋮19: **end for**20:**end for**21:**return** $d_{1}$, ⋯, $d_{m}$, $d_{P}$

## 5. Numerical Results

**Figure 5:**
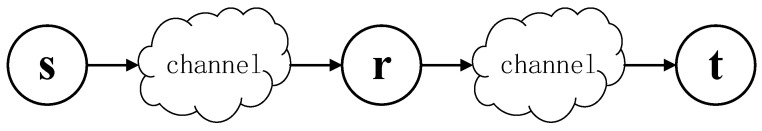
A two-hop line-erasure network with packet erasure probability 0.2 on each link.

In this section, we present some simulation results to illustrate the performance of our proposed coding scheme. As is shown in Figure 5, we consider the two-hop line-erasure network with one relay node **r** between the source **s** and destination **t** where the channel erasure probability of each hop is 0.2.

Unless explicitly stated, we use the following parameters: the number of source packets is K=10,000 where MIP occupies 0.1, the weight factor of MIP is $T_{1}=2$, and the size of a batch is M=16. The channel rank distribution and degree distribution of the BATS code are shown in Table 1 and Table 2 [3], respectively.

We set γ=0.45 and R=0.95. Using Algorithm 1, we find that when $d_{C}=100$, the optimal degree distributions of precoding are $d_{1}=23$, $d_{2}=3$, $d_{P}=2$, while for $d_{C}=80$, they are $d_{1}=13$, $d_{2}=3$, $d_{P}=2$. We randomly generate the precoding check matrix $H=\left[\begin{array}{cc} H_{1} & H_{2}|H_{P} \end{array}\right]$ according to the degree distribution, where $H_{P}$ is a non-singular matrix.

**Figure 6:**
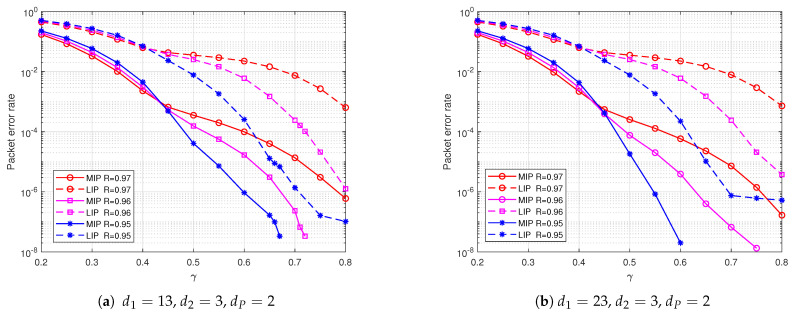
The packet error probability of weighted BATS codes based on PR-LDPC precoding at different precoding code rates.

Figure 6 shows the packet error rate of the weighted BATS code based on PR-LDPC precoding at different precoding rates. From the simulation results, it can be observed that when the coding redundancy γ is small (γ<0.45), the packet error rate of using precoding at different rates is very close. Because with a high-rate precoding, the receiver mainly relies on the weighted BATS decoder to recover the input packets, while the precoding decoder almost does not recover input packets. Therefore, the performance of using different precoding rates is similar to that of the conventional weighted BATS code in this case. When the coding redundancy is small, the packet error rate slightly increases as the precoding rate decreases. Because under the condition of constant coding redundancy, lower precoding rates introduce more redundancy in the precoding stage and less redundancy in the weighted BATS code stage. Since the decoding error probability mainly depends on the performance of the weighted BATS code when the coding redundancy is small, the lower precoding rates result in higher overall packet error rate. However, as the coding redundancy γ increases and the receiver recovers enough input packets, the number of input packets recovered in the precoding decoding stage increases. Moreover, the lower the precoding rate, the more input packets are recovered in the precoding decoder. Thus, the packet error rate decreases faster, and the error floor is lower.

**Figure 7:**
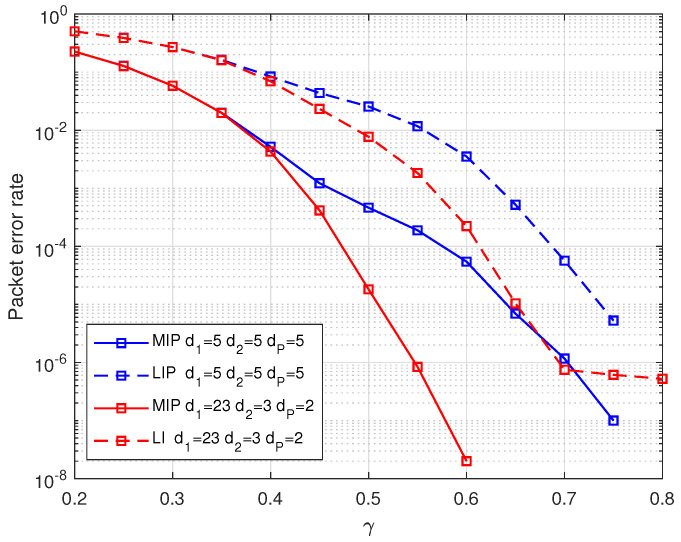
The packet error rate of weighted BATS codes based on PR-LDPC precoding with different precoding degree distributions.

Figure 7 demonstrates the performance of weighted BATS codes with PR-LDPC precoding using various degree distributions in terms of packet error rate. The simulation results indicate that when the encoding redundancy is small, the packet error rate is nearly identical across different precoding degree distributions. This can be attributed to the fact that, at this stage, the decoding of the weighted BATS codes is primarily responsible for recovering input packets. However, as the encoding redundancy increases, the packet error rate is highest when utilizing regular LDPC codes as precoding. This is due to the higher degree of LIP in regular LDPC codes with the same check node degree, and the higher error rate of LIP. As a result, in BP decoding, the probability of deleting edges connected to check nodes with LIP is lower, resulting in a lower probability of check nodes with degree 1. Consequently, the packet error rate is higher when employing regular LDPC codes as precoding.

**Figure 8:**
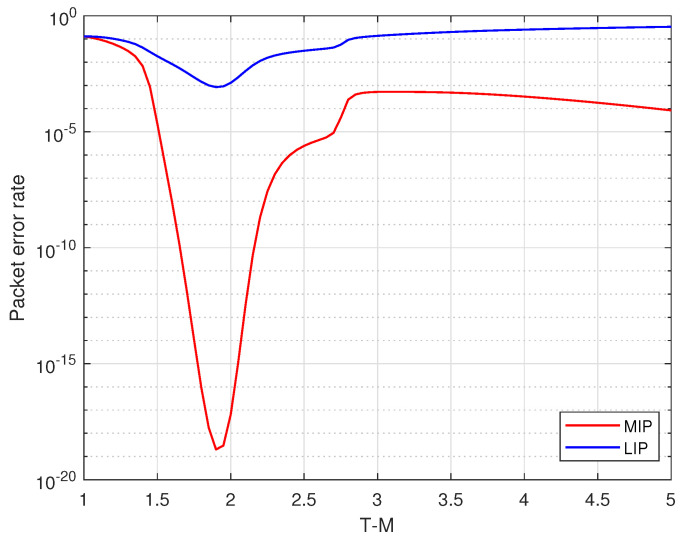
The packet error rate of proposed scheme with different $T_{M}$ when γ=0.48.

**Figure 9:**
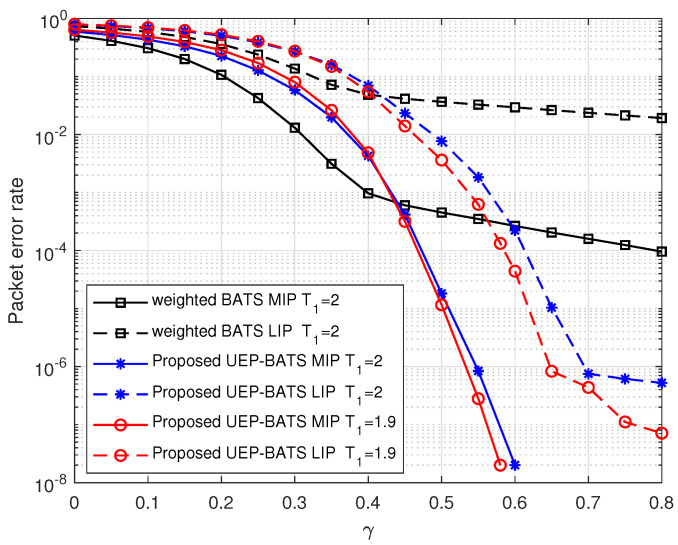
The packet error rate comparison of the proposed coding scheme and conventional weighted BATS codes with different encoding redundancy γ when R=0.95.

Given the precoding degree distribution and γ, we can optimize $T_{M}$ with the DE analysis in Sec. III B. Figure 8 shows the performance of MIP and LIP with different $T_{1}$ when γ=0.48 and precoding degree distribution is $d_{1}=23$, $d_{2}=3$, $d_{P}=2$. According to the asymptotic results, the error probability of MIP is minimized when $T_{1}=1.9$. The simulation result in Figure 9 shows the packet error rate of weighted BATS codes and our proposed scheme when $T_{1}=1.9$ and $T_{1}=2$. As is shown in Figure 9, when the coding redundancy is small, the error rate of our proposed scheme is slightly higher than that of the weighted BATS codes. That is because precoding can hardly lower the packet error rate but increases the overall encoding redundancy. As the coding redundancy increases, the conventional weighted BATS codes have high error floor. The packet error rate of our proposed scheme drops sharply, which is significantly lower than that of the conventional weighted BATS codes. Meanwhile, in our scheme, the packet error rate when $T_{1}=1.9$ is lower than that when $T_{1}=2$, which verifies the optimization results above.

## 6. Conclusions

In this paper, we propose a novel UEP-BATS coding scheme which performs UEP-LDPC precoding before the weighted BATS code. Compared with conventional weighted BATS codes, our proposed coding scheme has lower packet error rate. We analyze the situation where the input packets have two important levels in detail and derive the DE formulas of the proposed coding scheme decoded using the BP algorithm on BEC. We also optimize precoding degree distribution and weight factor of weighted BATS with the help of DE analysis. Finally, simulation results show that compared with the conventional weighted BATS code, our coding scheme has lower packet error rate with the increase of encoding redundancy.

## Figures and Tables

**Table 1:** The rank distribution of the two-hop erasure network when channel erasure probability is 0.2.

r	1	2	3	4	5	6	7	8
$h_{r}$	0	0	0	0	0.0001	0.0004	0.0025	0.0110
r	9	10	11	12	13	14	15	16
$h_{r}$	0.0387	0.1040	0.2062	0.2797	0.2338	0.1038	0.0190	0.0008

**Table 2:** The BATS codes degree distribution when M = 16.

d	14	15	20	21	28	38	39
$\Omega_{d}$	0.0478	0.2665	0.1012	0.0977	0.1411	0.0899	0.0122
d	51	52	73	74	111	113	199
$\Omega_{d}$	0.0034	0.0734	0.0579	0.0061	0.0251	0.0286	0.0491

## Data Availability

No new data were created or analyzed in this study. Data sharing is not applicable to this article.
